# Supplementary material for: Co-evolved Partners of Immunity: A Trait-Based Map of Human Keystone Organisms
Source: bioRxiv. 2025 Dec 30:2025.08.19.671142. Preprint. [Version 2] doi: 10.1101/2025.08.19.671142 (PMC12773006; doi:10.1101/2025.08.19.671142)
Supplement: Supplementary file 1 [file NIHPP2025.08.19.671142v2-supplement-2.pdf]

bioRxiv preprint doi: https://doi.org/10.1101/2025.08.19.671142; this version posted December 30, 2025. The copyright holder for this preprint (which was not certified by peer review) is the author/funder, who has granted bioRxiv a license to display the preprint in perpetuity. It is made available under aCC-BY 4.0 International license.

A Summary Tables of Pathogen Characteristics

Tables 3–6 provide detailed immunological and evolutionary characteristics for each pathogen, organized by archetype assignment. For each organism, we document infection pattern, geographic distribution, key immune dependencies, capacity for lifelong infection, primary tissues and cell types involved, clinical impact under immunosuppression, and estimated co-evolutionary time with humans.

bioRxiv preprint doi: https://doi.org/10.1101/2025.08.19.671142; this version posted December 30, 2025. The copyright holder for this preprint (which was not certified by peer review) is the author/funder, who has granted bioRxiv a license to display the preprint in perpetuity. It is made available under aCC-BY 4.0 International license.

Table 3 Keystone organisms and their immunological and evolutionary characteristics. These pathogens exhibit multi-arm immune coordination, structured tissue tropism, and deep co-evolution with humans. Reactivation in immunosuppressed hosts reflects systems-level failure. Abbreviations in parentheses match those used in Figures and trait score tables.

**Organism (Abbr.)**	**Type**	**Infection Pattern**	**Geo. Dist.**	**Key Immune Dependencies**	**Lifelong?**	**Tissues/Cell Involved**	**Immunosuppression Impact**	**Co-evo. Time**
Cytomegalovirus (CMV)	Herpesvirus	Chronic latency	Global	NK, $CD8^+$, $CD4^+$, Abs	Yes	Monocytes/Macrophages, Endothelium	Post-transplant reactivation; severe disease with T/NK suppression	5–25 Mya
Epstein–Barr Virus (EBV)	Herpesvirus	Chronic latency	Global	$CD8^+$, NK; $CD4^+$ help	Yes	Memory B cells, Oropharyngeal epithelium	PTLD with B-cell depletion; risk with $CD8^+$ defects	12–14 Mya
Herpes Simplex Virus 1 (HSV-1)	Herpesvirus	Chronic latency	Global	$CD8^+$ (TRM), NK; $CD4^+$ help	Yes	Mucosal epithelium, Sensory neurons	Severe mucocutaneous/CNS disease in NK or $CD8^+$ defects	∼6 Mya
Herpes Simplex Virus 2 (HSV-2)	Herpesvirus	Chronic latency	Global	$CD8^+$, NK; $CD4^+$ help	Yes	Genital mucosa, Sensory neurons	Severe genital/CNS disease in NK or $CD8^+$ defects	<1.6 Mya
Human Herpesvirus 6 (HHV-6)	Herpesvirus	Chronic latency	Global	$CD4^+$, $CD8^+$, NK	Yes	$CD4^+$ T cells, CNS	Encephalitis/reactivation post-transplant; risk with $CD4^+$ loss	∼30 Mya
Mycobacterium tuberculosis (Mtb)	Bacteria	Intracellular persistence	Global	$CD4^+$, TNF, IFN-γ	Yes	Alveolar macrophages, Granulomas	Reactivation with TNF blockade or $CD4^+$ loss	0.07–3 Mya
Varicella–Zoster Virus (VZV)	Herpesvirus	Chronic latency	Global	$CD4^+$, $CD8^+$ (TRM)	Yes	Dorsal root ganglia, Skin	Zoster with aging or T-cell suppression; dissemination on high-dose steroids	25–30 Mya

bioRxiv preprint doi: https://doi.org/10.1101/2025.08.19.671142; this version posted December 30, 2025. The copyright holder for this preprint (which was not certified by peer review) is the author/funder, who has granted bioRxiv a license to display the preprint in perpetuity. It is made available under aCC-BY 4.0 International license.

Table 4 Specialist organisms and their immunological and evolutionary characteristics. These pathogens show focused tropism and epitope-centric strategies with moderate coordination, often maintaining chronic infection without broad systems-level imprinting. Abbreviations in parentheses match those used in Figures and trait score tables.

Organism (Abbr.)	Type	Infection Pattern	Geo. Dist.	Key Immune Deps.	Lifelong?	Tissues/Cell Involved	Immunosuppression Impact	Co-evo. Time
BK Virus (BKV)	Polyomavirus	Chronic latency	Global	$CD8^+$, $CD4^+$, Abs	Yes	Renal tubular epithelium	BK nephropathy post-transplant; risk with T-cell dysfunction	>400 kya
Candida albicans (C.alb)	Fungus	Colonization/ overgrowth	Global	Th17, Neutrophils, Macrophages	Yes	Oral/genital mucosa, Skin	Mucocutaneous disease with IL-17 blockade or steroids	>20 Mya
Helicobacter pylori (H.pyl)	Bacteria	Chronic colonization	Global	Th1/Th17; B cells	Yes	Gastric epithelium	Persistent colonization; activity shaped by T-cell/IL-17 pathways	60–100 kya
Human Papillomavirus (HPV)	Papillomavirus	Chronic latency	Global	$CD8^+$, $CD4^+$, NK	Yes	Basal epithelial cells (anogenital/oropharyngeal)	High-grade lesions with T-cell defects; transplant risk	~500 kya
JC Virus (JCV)	Polyomavirus	Chronic latency	Global	$CD4^+$, $CD8^+$, IFN-γ	Yes	Oligodendrocytes, Astrocytes	PML with $CD4^+$ loss or integrin blockade	400–500 kya
Kaposi's Sarcoma-Assoc. Herpesvirus (KSHV)	Herpesvirus	Chronic latency	Focal	$CD8^+$, NK; $CD4^+$ help	Yes	Endothelium, B cells	KS/viremia in HIV/-transplant; T-cell suppression	20–30 Mya
Merkel Cell Polyomavirus (MCPyV)	Polyomavirus	Chronic latency	Global	$CD8^+$, $CD4^+$, IFN-γ	Yes	Merkel cells, Skin	Merkel cell carcinoma risk with T-cell suppression	>500 kya
Neisseria meningitidis (N.men)	Bacteria	Colonization + invasion	Global	Complement (C5–9), B cells	No	Nasopharynx, Meninges	Invasive disease with complement deficiency/asplenia	500–1000 kya
Parvovirus B19 (PVB19)	Virus	Seasonal infection	Global	Neutralizing Abs; $CD8^+$	No	Erythroid progenitors (marrow)	Chronic anemia in immunodeficiency/-transplant	10–20 kya
Pneumocystis jirovecii (P.jir)	Fungus	Chronic colonization	Global	$CD4^+$, Macrophages	No	Alveoli (type I/II interface)	PCP with $CD4^+$ loss (AIDS, steroids)	100–200 kya
Treponema pallidum (T.pal)	Bacteria	Mucosal contact	Global	$CD4^+$, Macrophages	No	Skin/mucosa, Endothelium, CNS	Neurosyphilis/relapses with T-cell suppression	2.5–15 Mya

bioRxiv preprint doi: https://doi.org/10.1101/2025.08.19.671142; this version posted December 30, 2025. The copyright holder for this preprint (which was not certified by peer review) is the author/funder, who has granted bioRxiv a license to display the preprint in perpetuity. It is made available under aCC-BY 4.0 International license.

Table 5 Multi-hostorganisms with broad tissue engagement but lacking deep co-evolution, human specificity, or stable imprinting. They can trigger strong immune activation or mimicry yet remain evolutionarily unsettled. Abbreviations in parentheses match those used in Figures and trait score tables.

Organism (Abbr.)	Type	Infection Pattern	Geo. Dist.	Key Immune Deps.	Lifelong?	Tissues/Cell Involved	Immunosuppression Impact	Co-evo. Time
Aspergillus spp. (Asper)	Fungus	Airborne inhalation	Ubiquitous	Neutrophils, Macrophages, $CD4^+$	No	Lungs, Paranasal sinuses; CNS (disseminated)	Invasive disease with neutropenia or high-dose steroids	<10 kya
Cryptococcus neoformans (C.neo)	Fungus	Environmental exposure	Global	$CD4^+$, Macrophages	Yes	Lungs, CNS (meningitis)	Severe disease in $CD4^+$ loss; transplant risk	Millions (environmental)
Histoplasma capsulatum (H.cap)	Fungus	Inhalation	Endemic (Americas)	Macrophages, $CD4^+$, IFN-γ	Yes	Lungs, RES (liver, spleen)	Dissemination with $CD4^+$/TNF blockade	<10 kya
Leishmania spp. (Leish)	Protozoan	Vector-borne	Trop/ Subtrop	Macrophages, IFN-γ, TNF	Yes	Skin, Liver/Spleen (RES)	Reactivation with TNF/IFN-γ suppression	>15 Mya (clade)
Mycobacterium avium complex (MAC)	Bacteria	Opportunistic	Global	Macrophages, $CD4^+$	No	Lungs, RES	Dissemination with $CD4^+$ loss (AIDS)	<10 kya
Plasmodium falciparum (P.fal)	Protozoan	Chronic infection	Trop/Subtrop	$CD4^+$, IFN-γ, Abs	No	Hepatocytes, RBCs, Microvasculature	Severe disease with impaired $CD4^+$ responses; pregnancy risk	50–100 kya
Schistosoma spp. (Schis)	Helminth	Chronic infection	Trop/ Subtrop	Th2, Eosinophils, Macrophages	Yes	Mesenteric/vesical veins, Liver, GI	Granulomas; immune remodeling; steroid effects	1–4 Mya
Strongyloides stercoralis (S.ste)	Helminth	Auto-infection	Trop/ Subtrop	Th2, Eosinophils	Yes	GI tract, Lungs	Hyperinfection on steroids; HTLV-1 co-risk	20–50 kya
Toxoplasma gondii (T.gon)	Protozoan	Food/zoonotic	Global	$CD8^+$, $CD4^+$, IFN-γ	Yes	CNS, Retina, Muscle	Encephalitis/retinitis with $CD4^+$ loss	∼10 kya (domestication)
Trypanosoma cruzi (T.cru)	Protozoan	Vector-borne	Latin America	$CD8^+$, $CD4^+$, IFN-γ	Yes	Cardiac muscle, GI, CNS	Reactivation in AIDS/transplant	∼9 kya

bioRxiv preprint doi: https://doi.org/10.1101/2025.08.19.671142; this version posted December 30, 2025. The copyright holder for this preprint (which was not certified by peer review) is the author/funder, who has granted bioRxiv a license to display the preprint in perpetuity. It is made available under aCC-BY 4.0 International license.

Table 6 Opportunistic organisms characterized by low coordination, environmental ubiquity, and narrow immune dependencies. Their emergence under immunosuppression flags pathway-specific failure rather than systemic collapse. Abbreviations in parentheses match those used in Figures and trait score tables.

Organism (Abbr.)	Type	Infection Pattern	Geo. Dist.	Key Immune Deps.	Lifelong?	Tissues/Cell Involved	Immunosuppression Impact	Co-evo. Time
Acanthamoeba spp. (Acan)	Amoeba	Contact/ Inhalation	Global (soil, water)	$CD4^+$, Macrophages	No	Cornea, CNS	Keratitis/CNS disease with T-cell defects	Unknown
Balamuthia mandrillaris (B.man)	Amoeba	Contact/ Inhalation	Soil, water	$CD4^+$	No	CNS, Skin	Granulomatous amebic encephalitis in T-cell suppression	Unknown
Bartonella spp. (Barto)	Bacteria	Vector-borne	Ubiquitous (cats, lice)	$CD4^+$, Macrophages	Yes	Endothelium, Erythrocytes	Bacillary angiomatosis with T-cell suppression	∼10 kya
Brucella spp. (Bruce)	Bacteria	Zoonotic	Livestock-associated	$CD4^+$, IFN-γ, Macrophages	Yes	RES, Reproductive tract	Chronic infection with TNF/IFN-γ axis defects	<10 kya
Burkholderia cepacia complex (Bcc)	Bacteria	Contact/ Inhalation	Water, hospitals	Neutrophils; oxidative burst	No	Lungs, Bloodstream	Severe in CGD/CF; neutropenia risk	<10 kya
Coccidioides spp. (Cocci)	Fungus	Inhalation	Endemic (US SW)	$CD4^+$, Macrophages	No	Lungs, CNS	Dissemination with $CD4^+$ loss	<10 kya
Coxiella burnetii (C.bur)	Bacteria	Inhalation	Global (dust/aerosol)	IFN-γ, TNF, Macrophages	No	Lungs, Liver, Endothelium	Severe disease with TNF/IFN-γ blockade	<10 kya
Fusarium spp. (Fusar)	Fungus	Inhalation/ Direct	Global (vegetation)	Neutrophils, Macrophages	No	Skin, Lungs, Blood	Dissemination with neutropenia/steroids	<10 kya
Human Herpesvirus 7 (HHV-7)	Herpesvirus	Chronic latency	Global	$CD4^+$, NK	Yes	T cells ($CD4^+$)	Reactivation with T-cell suppression	25–30 Mya
Legionella pneumophila (L.pne)	Bacteria	Inhalation	Water systems	IFN-γ, Macrophages	No	Lungs, Macrophages	Severe pneumonia with corticosteroids or TNF blockade	<100 years
Leptospira spp. (Lepto)	Bacteria	Zoonotic contact	Trop/Subtrop	Neutrophils, Macrophages, Abs	No	Kidney, Liver, Endothelium	Severe leptospirosis with innate/B-cell defects	5–10 kya
Nocardia spp. (Nocar)	Bacteria	Inhalation	Global (soil)	$CD4^+$, Macrophages	No	Lungs, Brain, Skin	Dissemination with T-cell suppression	<10 kya
Scedosporium spp. (Scedo)	Fungus	Inhalation	Global (stagnant water)	Neutrophils, Macrophages	No	Lungs, CNS	Severe disease with neutropenia; transplant risk	<10 kya
Torque Teno Virus (TTV)	Virus	Ubiquitous viremia	Near-universal	Tracks T-cell status (biomarker)	No	Blood, Lymphocytes	Higher loads with T-cell suppression; biomarker of net state	>500 kya
Vibrio spp. (Vibrio)	Bacteria	Ingestion/ Wound	Coastal/ Estuarine	Neutrophils, Complement	No	GI tract, Skin; Liver disease risk	Severe disease with liver disease or complement defects	<10 kya

bioRxiv preprint doi: https://doi.org/10.1101/2025.08.19.671142; this version posted December 30, 2025. The copyright holder for this preprint (which was not certified by peer review) is the author/funder, who has granted bioRxiv a license to display the preprint in perpetuity. It is made available under aCC-BY 4.0 International license.

Table 7 Trait definitions and score interpretations used to assess keystoneness.

**Trait**	**Allowed Scores**	**Score Interpretation**
Human-specific	{0,1}	1: Sustained human-only transmission.<br>0: Multi-host or zoonotic cycle.
Latency and Lifelong Infection	{0,1,2,3}	3: Lifelong latency with immune calibration.<br>2: Common persistence.<br>1: Limited or sporadic persistence. 0: None.
Length of Co-evolution with Humans	{0,1,2,3}	3: Deep-time co-divergence. 2: Long-standing association.<br>1: Recent emergence. 0: Recent zoonosis.
Global Prevalence	{0,1,2,3}	3: High global burden. 2: Intermediate.<br>1: Low or regional. 0: Rare.
Genetic Polymorphisms Reflecting Human Migration	{0,1,2,3}	3: Pathogen lineages tightly track human migration.<br>2: Partial coupling. 1: Contextual. 0: None.
Prevalence in Modern Indigenous Populations	{0,1,2,3}	3: High prevalence in multiple isolated populations.<br>2: Replicated across groups. 1: Limited. 0: None.
Consequences of Delayed Acquisition	{0,1,2,3}	3: Delayed infection causes severe disease.<br>2: Common age-related effects.<br>1: Context-specific. 0: None.
Association with Transplant Rejection	{0,1,2,3}	3: Robust, replicated clinical link.<br>2: Plausible but mixed. 1: Limited. 0: None.
Cross-reactivity with Adaptable RNA Viruses	{0,1}	1: Documented heterologous immunity or cross-reactivity.<br>0: None.
Perturbation by RNA Viruses	{0,1,2,3}	3: Frequent reactivation/modulation by RNA viruses.<br>2: Repeated in defined contexts.<br>1: Sporadic reports. 0: None.
Hypersensitivity and Autoimmune Flares	{0,1,2,3}	3: Reproducible links to autoimmune flares or immune complex disease.<br>2: Multiple plausible reports. 1: Sparse. 0: None.
Structured Immune Receptor Selection	{0,1,2,3}	3: Public or stereotyped TCR/BCRs with function.<br>2: Convergent motifs with partial function.<br>1: Scattered hints. 0: None.
Proximity and Persistence of Immune Cells	{0,1,2,3}	3: TRM or TLS consistently present with systemic effects.<br>2: Frequent persistence with limited scope.<br>1: Sparse. 0: None.
Epitope Structure and Immunodominance	{0,1,2,3}	3: Reproducible dominant epitopes across cohorts.<br>2: Replicated patterns. 1: Limited. 0: None.
Absence of Peripheral Tolerance	{0,1,2,3}	3: Established mimicry with autoimmune outcomes.<br>2: Strong evidence of tolerance break.<br>1: Suggestive hints. 0: None.
Epitope Presentation Dynamics	{0,1,2,3}	3: Strong HLA associations or MHC modulation.<br>2: Replicated effects. 1: Limited. 0: None.
Multi-Arm Coordination	{0,1}	1: Coordinated innate–adaptive containment with redundancy.<br>0: Partial or local coordination only.
Systemic Tropism	{0,1}	1: Multi-niche or systemic tissue persistence.<br>0: Localized or stochastic tropism.

bioRxiv preprint doi: https://doi.org/10.1101/2025.08.19.671142; this version posted December 30, 2025. The copyright holder for this preprint (which was not certified by peer review) is the author/funder, who has granted bioRxiv a license to display the preprint in perpetuity. It is made available under aCC-BY 4.0 International license.

B Trait-Based Scoring: Technical Details

B.1 Score vector representation

Each pathogen i was represented by an 18-dimensional score vector $\mathbf{x}_i = (x_{i,1}, \ldots, x_{i,18})$, where $x_{i,t}$ denotes the score for pathogen i on trait t. Total keystoneness scores were computed as unweighted sums:

$$S_i = \sum_{t=1}^{18} x_{i,t}$$

B.2 Gower distance

Pairwise dissimilarities were computed using Gower distance, which accommodates mixed ordinal and binary data while handling missing values:

$$d_{ij} = \frac{\sum_{t=1}^{p} s_{ijt}\,\delta_{ijt}}{\sum_{t=1}^{p} s_{ijt}}$$

where $s_{ijt} \in \{0, 1\}$ indicates whether trait t is observed for both pathogens i and j (non-missingness indicator), and δ_{ijt} is the trait-specific dissimilarity:

$$\delta_{ijt} = \begin{cases} |x_{i,t} - x_{j,t}|/R_t, & \text{if trait } t \text{ is ordinal} \\ \mathbb{1}\{x_{i,t} \neq x_{j,t}\}, & \text{if trait } t \text{ is binary} \end{cases}$$

Here R_t is the observed range for ordinal trait t, and $\mathbb{1}\{\cdot\}$ is the indicator function.

B.3 Clustering algorithms

Two clustering approaches were applied to the Gower distance matrix:

Partitioning around medoids (PAM): PAM minimizes the sum of dissimilarities between each object and its assigned medoid:

$$\min_{m_1,\ldots,m_k} \sum_{i=1}^{n} \min_{j\in\{1,\ldots,k\}} d_{i,m_j}$$

where $m_1, \ldots, m_k$ are the indices of the k medoid organisms.

Hierarchical clustering: Agglomerative clustering with complete linkage, where the distance between clusters is defined as the maximum pairwise distance:

$$D(A, B) = \max_{i\in A, j\in B} d_{ij}$$

bioRxiv preprint doi: https://doi.org/10.1101/2025.08.19.671142; this version posted December 30, 2025. The copyright holder for this preprint (which was not certified by peer review) is the author/funder, who has granted bioRxiv a license to display the preprint in perpetuity. It is made available under aCC-BY 4.0 International license.

B.4 Cluster number selection

The number of clusters was selected using elbow and angle heuristics applied to within-cluster sum of dissimilarities $W(k)$ for $k = 2, \ldots, 8$:

$$W(k) = \sum_{c=1}^{k} \sum_{i \in C_c} d_{i,m_c}$$

Both methods nominated $k = 4$ clusters.

B.5 Consensus clustering

Method agreement was quantified by the Adjusted Rand Index (ARI):

$$\text{ARI} = \frac{\text{RI} - \mathbb{E}[\text{RI}]}{\max(\text{RI}) - \mathbb{E}[\text{RI}]}$$

where RI is the Rand Index measuring pairwise agreement between clusterings.

For organisms with discordant assignments between PAM and hierarchical clustering, we applied a conservative consensus rule: each organism was assigned to the candidate cluster with the lower mean total score $\bar{S}_c$. Four organisms were reassigned under this rule, including Parvovirus B19 (Keystone $\rightarrow$ Specialist) and three additional organisms (Specialist $\rightarrow$ Opportunist). To further enhance the specificity of the keystone label, organisms initially assigned to the keystone archetype underwent an additional centroid-based validation step and were reassigned if their total trait score was closer to the centroid of another cluster.

B.6 Trait signature analysis

Cluster-versus-rest comparisons used Cliff's delta (δ) for effect size, defined as:

$$\delta = \frac{\#(x_i > y_j) - \#(x_i < y_j)}{n_1 n_2}$$

where x_i are scores in the focal cluster and y_j are scores in remaining clusters. Wilcoxon rank-sum tests were used for significance testing. Statistical significance was assessed using uncorrected p-values at $\alpha = 0.05$ (*** $p < 0.001$, ** $p < 0.01$, * $p < 0.05$).

Multivariate signatures were obtained from ℓ_1-penalized (LASSO) multinomial logistic regression:

$$\hat{\boldsymbol{\beta}} = \arg\min_{\boldsymbol{\beta}} \left\{ -\ell(\boldsymbol{\beta}) + \lambda \sum_{c,t} |\beta_{ct}| \right\}$$

where $\ell(\boldsymbol{\beta})$ is the multinomial log-likelihood, the ℓ_1 penalty encourages sparsity in trait selection, and λ was selected by 10-fold cross-validation. The regularization parameter $\alpha = 1$ specifies pure LASSO penalization.

bioRxiv preprint doi: https://doi.org/10.1101/2025.08.19.671142; this version posted December 30, 2025. The copyright holder for this preprint (which was not certified by peer review) is the author/funder, who has granted bioRxiv a license to display the preprint in perpetuity. It is made available under aCC-BY 4.0 International license.

C Clinical Emergence Tensor: Scoring and Analysis

We constructed a three-dimensional clinical emergence tensor $\mathbf{T} \in \mathbb{R}^{43\times13\times31}$ documenting pathogen reactivation or invasion patterns across:

- 43 pathogens (matching those in the immunological trait analysis)
- 13 anatomical niches (blood/systemic, CNS, respiratory mucosa, GI mucosa, genitourinary mucosa, skin, reticuloendothelial system, bone marrow, lymph nodes, cardiac/endovascular, bone/joint, ocular, lymph node germinal centers)
- 31 immune perturbations spanning genetic immunodeficiencies and pharmacologic immunosuppression

C.1 Scoring Rubric

C.1.1 Tensor construction and scoring

Each tensor element $T(p, n, i) \in [0, 100]$ represents the strength of clinical evidence for lytic emergence, scored according to:

- **85–100**: Replicated human studies (genetic or interventional), hallmark associations
- **70–84**: Solid human signal for this niche with multiple sources or one high-quality source
- **40–69**: Clear but context-dependent human evidence, dose/timing dependent, partial compensation by other immune arms
- **10–39**: Suggestive or narrow human signals, or strong mechanistic logic with limited human data
- **5**: No authoritative human evidence found after diligent search
- **0**: Published evidence indicates protective effect or no increased emergence

The threshold at 40 demarcates human-relevant clinical data (scores ≥ 40 include at least limited human observations with mechanistic support, grade C or better) from preclinical evidence only (scores < 40, grade D).

C.1.2 Evidence grading and confidence annotation

Each score was accompanied by structured metadata:

- **Direction**: "revealed" (perturbation increases emergence), "protected" (reduces risk), or "unclear" (conflicting/insufficient data)
- **EvidenceGrade**:
  - A = replicated human genetic or interventional evidence
  - B = strong human clinical cohorts or case series
  - C = limited human data with strong mechanistic support
  - D = animal or in vitro evidence only
- **ConfidenceTag**: [High], [Moderate], [Low], [Minimal] (for score 5), [None] (for score 0)
- **Justification**: rigorous citations formatted as (Author, Title, Journal, Year)
- **EvidenceCount**: number of distinct human studies supporting the score

bioRxiv preprint doi: https://doi.org/10.1101/2025.08.19.671142; this version posted December 30, 2025. The copyright holder for this preprint (which was not certified by peer review) is the author/funder, who has granted bioRxiv a license to display the preprint in perpetuity. It is made available under aCC-BY 4.0 International license.

- **Missingness**: "observed" (evidence exists), "inferred_zero" (no evidence after diligent search), or "unknown"

Evidence was prioritized as: (1) human genetic/interventional perturbations with niche-localized outcomes, (2) human clinical cohorts with clear site localization, (3) human observations without precise site (stated with caveats), (4) animal/in vitro support only (capped at score 69, grade C or D).

C.1.3 Controlled vocabularies for niches and perturbations

Each anatomical niche was defined by controlled vocabularies specifying required anatomical terms (IncludeTokens) and exclusion criteria (ExcludeTokens) to ensure precise localization. For example, CNS required terms like "brain," "cerebral," "encephalitis," "CSF," or "meninges," while excluding "ocular" or "peripheral nerve." Similarly, each immune perturbation was defined by mechanism and synonyms. For instance, P07 (CalcineurInh) encompasses "tacrolimus," "cyclosporine," and "calcineurin inhibitor," all blocking NFAT and IL-2 signaling. Complete niche and perturbation definitions are in Supplementary Section C.2.

C.1.4 Sensitivity analyses: Score adjustment schemes

To assess robustness to evidence quality, we performed sensitivity analyses by adjusting scores based on evidence grade and confidence:

Grade-capping.

Scores were reduced for lower evidence grades to prevent overweighting of animal/in vitro data:

- Grade A: score unchanged
- Grade B: score capped at 84 (if originally > 84)
- Grade C: score capped at 69 (if originally > 69)
- Grade D: score capped at 39 (if originally > 39)

Grade+Confidence weighting.

Scores were multiplicatively adjusted by a factor combining evidence grade and confidence tag, down-weighting low-confidence or low-grade entries. Weighting factors were:

- High: 1.0 (no reduction)
- Moderate: 0.85
- Low: 0.70
- Minimal/None: 0.50

Combined with grade-specific caps, this yielded conservative score estimates prioritizing high-quality human evidence.

All feature extraction (max-marginalization, breadth metrics), classification (elastic-net regression), and clustering (PAM) analyses were re-run on both adjusted

bioRxiv preprint doi: https://doi.org/10.1101/2025.08.19.671142; this version posted December 30, 2025. The copyright holder for this preprint (which was not certified by peer review) is the author/funder, who has granted bioRxiv a license to display the preprint in perpetuity. It is made available under aCC-BY 4.0 International license.

score tensors to test whether archetype predictions and unsupervised recovery remain robust when evidence quality is explicitly down-weighted.

C.2 Tensor Dimensions

The clinical tensor $\mathbf{T} \in \mathbb{R}^{43\times13\times31}$ captures pathogen behavior across anatomical niches and immune perturbations. Below we provide detailed definitions for each dimension.

C.2.1 Anatomical Niches (N=13)

1. **CNS (Central Nervous System)**: Brain parenchyma, meninges, cerebrospinal fluid spaces. Reflects capacity for neuroinvasion, meningitis, or encephalitis. Relevant for neurovirulent pathogens and those causing CNS opportunistic infections.
2. **Lung (Pulmonary)**: Pulmonary parenchyma, airways, alveolar spaces. Encompasses pneumonia, bronchitis, and respiratory tract colonization. Central niche for respiratory viruses, bacteria, and fungi.
3. **GI (Gastrointestinal)**: Gastrointestinal mucosa, lumen, and associated lymphoid tissue (GALT). Includes enteric infections, gastroenteritis, and mucosal colonization. Relevant for fecal-oral transmission routes.
4. **Liver (Hepatic)**: Hepatic parenchyma, including hepatocytes, Kupffer cells, and bile ducts. Reflects hepatotropism, hepatitis, and intrahepatic replication. Important for bloodborne and vector-borne pathogens.
5. **Blood (Bloodstream/Intravascular)**: Intravascular space, including circulating immune cells and endothelium. Captures bacteremia, viremia, parasitemia, and disseminated infections. Critical for sepsis-causing organisms.
6. **LN_Paracortex (Lymph Node T Cell Zones)**: T cell-rich paracortical regions of lymph nodes. Site of adaptive T cell priming and cell-mediated immune responses. Reflects pathogens that manipulate or reside within T cell niches.
7. **LN_GC (Lymph Node Germinal Centers)**: B cell germinal centers within secondary lymphoid tissues. Sites of B cell maturation, somatic hypermutation, and antibody class-switching. Reflects pathogens that colonize or replicate within organized B cell zones.
8. **Spleen (Splenic)**: Splenic parenchyma, including red pulp (filtration) and white pulp (lymphoid). Central to bloodborne pathogen clearance and immune surveillance. Critical for encapsulated bacteria and blood-stage parasites.
9. **BoneMarrow (Hematopoietic Marrow)**: Bone marrow niches supporting hematopoiesis. Reflects pathogens causing marrow suppression, hemophagocytic syndromes, or intracellular marrow persistence.
10. **Skin (Cutaneous/Subcutaneous)**: Skin, subcutaneous tissue, and dermal immune cells. Includes cellulitis, abscesses, vector bite sites, and dermatological manifestations. Entry point for many vector-borne and traumatic infections.
11. **GU (Genitourinary)**: Genitourinary mucosa, renal parenchyma, bladder. Encompasses urinary tract infections, pyelonephritis, and sexually transmitted infections.

bioRxiv preprint doi: https://doi.org/10.1101/2025.08.19.671142; this version posted December 30, 2025. The copyright holder for this preprint (which was not certified by peer review) is the author/funder, who has granted bioRxiv a license to display the preprint in perpetuity. It is made available under aCC-BY 4.0 International license.

12. **Bone (Osseous)**: Bone tissue and periosteum. Reflects osteomyelitis, septic arthritis, and bone-invasive infections. Relevant for hematogenous seeding and trauma-associated infections.
13. **Eye (Ocular)**: Ocular tissues including conjunctiva, cornea, uvea, and retina. Captures keratitis, uveitis, endophthalmitis, and retinitis. Important for certain congenital and disseminated infections.

C.2.2 Immune Perturbations (N=31)

1. **P01 (Neutropenia)**: Severe neutrophil depletion (<500 cells/μL). Impairs bacterial and fungal killing, increasing risk for invasive bacterial and mold infections. Seen in chemotherapy, aplastic anemia, and congenital neutropenias.
2. **P02 (MacrophageDefect)**: Macrophage dysfunction or depletion. Compromises intracellular pathogen control, granuloma formation, and tissue clearance. Relevant for mycobacteria, Listeria, Salmonella, and intracellular parasites.
3. **P03 (CD4depletion)**: CD4+ T cell deficiency (e.g., HIV/AIDS, idiopathic CD4 lymphopenia). Central immunodeficiency affecting cell-mediated and humoral immunity. Predisposes to opportunistic infections and impaired vaccine responses.
4. **P04 (CD8depletion)**: CD8+ T cell deficiency or dysfunction. Impairs cytotoxic responses against intracellular pathogens and virally infected cells. Relevant for chronic viral infections and some intracellular bacteria.
5. **P05 (NKdepletion)**: Natural killer cell deficiency or dysfunction. Impairs early antiviral and antitumor responses. Associated with increased susceptibility to herpesviruses and some bacterial infections.
6. **P06 (TNFblock)**: TNF-α blockade via monoclonal antibodies (infliximab, adalimumab, etanercept). Impairs granuloma formation and macrophage activation. Major risk factor for tuberculosis reactivation and invasive fungal infections.
7. **P07 (IFNgBlock)**: IFN-γ neutralization or receptor deficiency. Critical for macrophage activation and intracellular pathogen control. Deficiency predisposes to disseminated mycobacterial and Salmonella infections.
8. **P08 (IL12p40block)**: IL-12/IL-23 p40 subunit blockade (ustekinumab). Impairs Th1 and Th17 responses. Increases risk for mycobacterial, fungal, and some bacterial infections.
9. **P09 (IL17block)**: IL-17A blockade (secukinumab, ixekizumab) or receptor deficiency. Impairs mucosal immunity and neutrophil recruitment. Predisposes to mucocutaneous candidiasis and some bacterial infections.
10. **P10 (IL1block)**: IL-1 blockade (anakinra, canakinumab) or receptor deficiency. Reduces pyogenic responses and fever. Modest infection risk, primarily pyogenic bacteria.
11. **P11 (C3deficiency)**: Complement C3 deficiency or depletion. Central complement component; deficiency severely impairs opsonization and immune complex clearance. High risk for encapsulated bacteria and Neisseria.
12. **P12 (ProperdinDeficiency)**: Properdin deficiency, stabilizer of alternative pathway C3 convertase. X-linked deficiency with markedly increased susceptibility to meningococcal disease.

bioRxiv preprint doi: https://doi.org/10.1101/2025.08.19.671142; this version posted December 30, 2025. The copyright holder for this preprint (which was not certified by peer review) is the author/funder, who has granted bioRxiv a license to display the preprint in perpetuity. It is made available under aCC-BY 4.0 International license.

13. **P13 (MBLdeficiency)**: Mannose-binding lectin deficiency. Impairs lectin pathway activation and opsonization. Modest increased risk for bacterial infections, particularly in young children.
14. **P14 (Hypogammaglobulinemia)**: Severe antibody deficiency (CVID, X-linked agammaglobulinemia). Impairs opsonization, neutralization, and immune complex clearance. High risk for encapsulated bacteria, enteroviruses, and chronic sinopulmonary infections.
15. **P15 (Bcelldepletion)**: B cell depletion via anti-CD20 therapy (rituximab, ocrelizumab) or congenital deficiency. Impairs antibody production and antigen presentation. Increases risk for bacterial infections and viral reactivation.
16. **P16 (TLRdefect)**: Toll-like receptor pathway defects (TLR signaling impairment). Reduces innate recognition of PAMPs. Variable susceptibility depending on specific TLR; broadly impairs innate immune priming.
17. **P17 (MyD88deficiency)**: MyD88 adaptor protein deficiency. Impairs most TLR and IL-1R signaling. Severe susceptibility to pyogenic bacteria, particularly Streptococcus pneumoniae and Staphylococcus aureus.
18. **P18 (IRAK4deficiency)**: IRAK4 kinase deficiency, downstream of MyD88. Similar phenotype to MyD88 deficiency with invasive bacterial infections in childhood.
19. **P19 (NFkBdefect)**: NF-κB pathway defects (NEMO, IκBα mutations). Impairs broad inflammatory and immune gene transcription. Variable phenotype with mycobacterial, bacterial, and fungal susceptibility.
20. **P20 (CGDdefect)**: Chronic granulomatous disease (phagocyte NADPH oxidase deficiency). Eliminates oxidative burst, impairing killing of catalase-positive organisms. High risk for Staphylococcus, Aspergillus, Burkholderia, Serratia, and Nocardia.
21. **P21 (MHC1deficiency)**: MHC class I deficiency (TAP deficiency, bare lymphocyte syndrome type I). Impairs CD8+ T cell antigen presentation. Increases susceptibility to viral infections.
22. **P22 (MHC2deficiency)**: MHC class II deficiency (bare lymphocyte syndrome type II). Impairs CD4+ T cell priming. Severe combined-like immunodeficiency with broad bacterial, viral, and fungal susceptibility.
23. **P23 (CD40Ldeficiency)**: CD40 ligand deficiency (X-linked hyper-IgM syndrome). Impairs T cell help for B cells and macrophage activation. Predisposes to Pneumocystis, Cryptosporidium, and bacterial infections.
24. **P24 (CTLA4agonist)**: CTLA-4 agonism or Ig fusion (abatacept). Inhibits T cell costimulation. Modest infection risk, primarily opportunistic and mycobacterial.
25. **P25 (PD1block)**: PD-1/PD-L1 blockade (checkpoint inhibitors: nivolumab, pembrolizumab). Enhances T cell activation; paradoxically can increase some infection risks via immune dysregulation and autoimmunity.
26. **P26 (IL6block)**: IL-6 or IL-6R blockade (tocilizumab, sarilumab). Reduces acute phase responses and Th17 differentiation. Increases risk for bacterial infections, diverticulitis, and some opportunistic pathogens.

bioRxiv preprint doi: https://doi.org/10.1101/2025.08.19.671142; this version posted December 30, 2025. The copyright holder for this preprint (which was not certified by peer review) is the author/funder, who has granted bioRxiv a license to display the preprint in perpetuity. It is made available under aCC-BY 4.0 International license.

27. **P27 (IL10block)**: IL-10 blockade or deficiency. IL-10 is anti-inflammatory; blockade may paradoxically enhance immunity but also cause inflammatory bowel disease. Included for completeness; rare clinical use.
28. **P28 (IL23block)**: IL-23 blockade (p19 subunit: guselkumab, risankizumab). More specific than IL-12/23 blockade; impairs Th17 responses. Infection risk appears lower than IL-12/23 blockade but includes fungal and mycobacterial concerns.
29. **P29 (JAKblock)**: JAK kinase inhibition (tofacitinib, baricitinib, upadacitinib). Broad impairment of cytokine signaling (IFN, IL-6, IL-12, IL-23, etc.). Increases risk for herpes zoster, tuberculosis, and opportunistic infections.
30. **P30 (CSFblock)**: GM-CSF or G-CSF blockade/deficiency. Impairs myeloid development, neutrophil function, and alveolar macrophage maturation. GM-CSF neutralization associated with pulmonary alveolar proteinosis and opportunistic lung infections.
31. **P31 (IL5block)**: IL-5 blockade via monoclonal antibodies (mepolizumab, reslizumab, benralizumab). IL-5 drives eosinophil development, survival, and activation. Blockade severely depletes eosinophils, impairing helminth immunity and potentially tissue eosinophil-mediated responses. Used clinically for severe eosinophilic asthma and hypereosinophilic syndrome.

C.3 Feature extraction from tensor and analysis

From the 3D tensor, we derived 2D feature matrices for each pathogen via max-marginalization:

$$X_{PI}(p,i) = \max_{n \in \text{niches}} T(p,n,i)$$
$$X_{PN}(p,n) = \max_{i \in \text{perturbations}} T(p,n,i)$$

These matrices capture the maximal emergence score across all contexts, yielding a 43×44 feature matrix per pathogen (31 immune perturbations + 13 niches).

We augmented this with breadth metrics:

$$\text{Immune breadth}(p) = \sum_{i=1}^{31} \mathbb{I}[X_{PI}(p,i) \geq 40]$$

$$\text{Niche breadth}(p) = \sum_{n=1}^{13} \mathbb{I}[X_{PN}(p,n) \geq 40]$$

quantifying the number of immune perturbations and anatomical niches exhibiting clinically significant emergence (threshold = 40, reflecting human-relevant data).

C.3.1 Supervised classification

We trained elastic-net logistic regression models to predict archetype membership from clinical features:

$$\hat{\boldsymbol{\beta}} = \arg\min_{\boldsymbol{\beta}} \left\{ -\ell(\boldsymbol{\beta}) + \lambda \left[\frac{1}{2}(1-\alpha)\|\boldsymbol{\beta}\|_2^2 + \alpha\|\boldsymbol{\beta}\|_1 \right] \right\}$$

bioRxiv preprint doi: https://doi.org/10.1101/2025.08.19.671142; this version posted December 30, 2025. The copyright holder for this preprint (which was not certified by peer review) is the author/funder, who has granted bioRxiv a license to display the preprint in perpetuity. It is made available under aCC-BY 4.0 International license.

where $\ell(\boldsymbol{\beta})$ is the log-likelihood (binomial for binary, multinomial for 4-way classification), $\alpha = 0.5$ balances ℓ_1 and ℓ_2 penalties, and λ was selected by cross-validation. We used 8-fold cross-validation for binary classification (keystone vs. non-keystone) to ensure adequate representation in each fold, and 5-fold for multi-class prediction to accommodate smaller archetype sample sizes. Models were fit using the `glmnet` package with AUC (binary) or misclassification error (multi-class) as the optimization criterion.

C.3.2 Unsupervised clustering validation

To test whether clinical emergence patterns independently recover trait-based archetypes, we performed PAM clustering on Gower dissimilarities of the feature matrix with $k = 4$ clusters. Cluster assignments were aligned to archetype labels via permutation optimization to maximize diagonal agreement, and concordance was quantified by Adjusted Rand Index (ARI). This unsupervised approach provides external validation that archetypes derived from immunological traits (Figure 4) correspond to distinct clinical emergence signatures.

All analyses (feature extraction, classification, clustering) were repeated for raw scores, grade-capped scores, and grade+confidence weighted scores to assess robustness across evidence quality thresholds.

bioRxiv preprint doi: https://doi.org/10.1101/2025.08.19.671142; this version posted December 30, 2025. The copyright holder for this preprint (which was not certified by peer review) is the author/funder, who has granted bioRxiv a license to display the preprint in perpetuity. It is made available under aCC-BY 4.0 International license.

D Mechanistic Model: Technical Details

D.1 Mechanistic scoring tables

Five expert-curated scoring tables quantified biological interactions (all scores normalized to $[0, 1]$):

1. **Latent control** $L(p, n_{\text{lat}}, c) \in [0, 1]$: Strength with which immune controller c suppresses pathogen p in latent niche n_{lat}. Higher scores indicate tighter immunological containment. (154 scored interactions)
2. **Lytic attack** $A(p, n) \in [0, 1]$: Capacity of pathogen p to breach barriers and cause clinical disease in niche n, independent of immune status. (145 scored interactions)
3. **Reactivation routes** $R(p, n_{\text{lat}} \to n_{\text{lytic}}) \in [0, 1]$: Probability that pathogen p escapes from latent reservoir n_{lat} to manifest clinically in lytic niche n_{lytic}. (151 scored interactions)
4. **Perturbation effects** $E(i, c) \in [0, 1]$: Degree to which immune perturbation i disables controller c. (144 scored interactions)
5. **Controller residency** $C(c, n) \in \{0, 1\}$: Binary indicator for whether controller c operates in niche n. (132 scored interactions)

D.1.1 Score normalization

Raw expert scores $s \in [0, 100]$ were normalized using:

$$s_{\text{norm}} = \begin{cases} \pi_{\text{unknown}} = 0.1 & \text{if } s = 5 \text{ (unknown)} \\ 0 & \text{if } s < 40 \text{ (biologically negligible)} \\ s/100 & \text{otherwise} \end{cases}$$

The threshold of 40 reflects expert consensus that lower scores indicate biologically negligible interactions. Crucially, unknown interactions (originally coded as 5) retain a small prior probability of 0.1 rather than being zeroed, reflecting that an unstudied interaction is not necessarily absent.

D.1.2 Perturbation effect scaling

Perturbation effects were scaled by qualitative effect type using multiplier κ:

- Depleted: $\kappa = 1.0$ (complete loss of controller)
- Disabled: $\kappa = 0.8$ (functional impairment with residual presence)
- Functionally impaired: $\kappa = 0.6$ (partial dysfunction)
- Unknown: $\kappa = 0.6$ (conservative default)

The final perturbation effect is $E_{\text{scaled}} = E_{\text{base}} \times \kappa$.

bioRxiv preprint doi: https://doi.org/10.1101/2025.08.19.671142; this version posted December 30, 2025. The copyright holder for this preprint (which was not certified by peer review) is the author/funder, who has granted bioRxiv a license to display the preprint in perpetuity. It is made available under aCC-BY 4.0 International license.

D.2 Latent pathway

The latent pathway models emergence through reactivation from immune-controlled reservoirs:

$$\text{Pathogen} \xrightarrow{L} \text{Latent Site} \xrightarrow{R} \text{Lytic Site} \xleftarrow{C} \text{Controller} \xleftarrow{E} \text{Perturbation}$$

For each mechanistic chain $(p, n_{\text{lat}}, n_{\text{lytic}}, c, i)$, the base score uses geometric mean (length-normalized):

$$s_{\text{base}} = \exp\left(\frac{1}{3}\sum_{x\in\{L,R,C\}} \log(x+\epsilon)\right)$$

where $\epsilon = 10^{-6}$ prevents $\log(0)$.

The perturbation effect acts as a modulatory exponent with strength $\beta = 1.5$:

$$s_{\text{chain}} = s_{\text{base}} \times E^{\beta}$$

This means complete controller disabling ($E = 1$) allows full expression of the base score, while partial effects attenuate emergence exponentially.

D.3 Barrier pathway

The barrier pathway models direct invasion when immune barriers are compromised:

$$\text{Pathogen} \xrightarrow{A} \text{Lytic Site} \xleftarrow{C} \text{Controller} \xleftarrow{E} \text{Perturbation}$$

To prevent over-triggering for controllers that don't actually control the pathogen, we added pathogen–controller specificity using latent control scores as a proxy:

$$PC(p, c) = \max_{n_{\text{lat}}} L(p, n_{\text{lat}}, c)$$

The barrier chain score is then:

$$s_{\text{barrier}} = A(p, n) \times E(i, c) \times C(c, n) \times PC(p, c)$$

D.4 Aggregation strategy

D.4.1 Collapse to best-per-controller

Multiple chains through the same controller are collapsed to avoid fake independence:

$$s_{\text{ctl}}(p, n, i, c) = \max_{\text{chains via } c} s_{\text{chain}}$$

bioRxiv preprint doi: https://doi.org/10.1101/2025.08.19.671142; this version posted December 30, 2025. The copyright holder for this preprint (which was not certified by peer review) is the author/funder, who has granted bioRxiv a license to display the preprint in perpetuity. It is made available under aCC-BY 4.0 International license.

D.4.2 Top-K selection

Only the top $K = 5$ controllers are retained before aggregation:

$$\mathcal{C}_{\text{top}}(p, n, i) = \text{top-}K\left(\{s_{\text{ctl}}(p, n, i, c)\}_c\right)$$

D.4.3 Normalized noisy-OR

Standard noisy-OR saturates quickly with multiple contributing causes. We use normalized noisy-OR:

$$\hat{T}_{\text{pathway}} = 1 - \prod_{c \in \mathcal{C}_{\text{top}}} (1 - s_{\text{ctl},c})^{1/K}$$

Property: If all K chains have score s, the result is exactly s (not ≈ 1).

D.4.4 Pathway combination

Latent and barrier pathways represent competing explanations, not additive probabilities:

$$\hat{T}_{\text{mix}}(p, n, i) = \max\left(\hat{T}_{\text{latent}}, \hat{T}_{\text{barrier}}\right)$$

D.5 Evaluation framework

D.5.1 The join direction problem

A critical methodological consideration: evaluation must start from the full observed tensor, not from predictions. Joining predictions to observations (rather than vice versa) would hide false negative and true negative mass, artificially inflating apparent performance. All evaluations use observation-first joins.

D.5.2 Binning to discrete rubric

Both predictions and observations were projected to coarse bins (multiples of 5: 0, 5, 10, . . . , 100) to match the discrete clinical scoring rubric:

$$\text{project}(x) = 5 \times \text{round}(x/5)$$

D.5.3 Three-way evaluation

Because zero clinical scores may represent either "true negative" or "not studied," we employed three complementary evaluation frameworks:

Evaluation 1: STRICT (all zeros = true negatives)

- Most conservative/pessimistic
- Treats every zero as evidence of absence
- Metrics: Pearson r, Spearman ρ, Cohen's weighted κ, F1, balanced accuracy

Evaluation 2: PU FRAMING (zeros = unlabeled)

bioRxiv preprint doi: https://doi.org/10.1101/2025.08.19.671142; this version posted December 30, 2025. The copyright holder for this preprint (which was not certified by peer review) is the author/funder, who has granted bioRxiv a license to display the preprint in perpetuity. It is made available under aCC-BY 4.0 International license.

- Positive-Unlabeled learning perspective
- Only positive observations are labeled; zeros are unknown
- Metrics: Precision@K, Recall@K, fold-enrichment

Fold-enrichment is defined as:

$$\text{Enrichment@}K = \frac{\text{Precision@}K}{\text{baseline rate}} = \frac{|\text{positives in top-}K|/K}{n_{\text{pos}}/N}$$

Evaluation 3: CONFIDENCE-MASKED (low-confidence zeros = missing)

- Uses evidence confidence to distinguish:
  - **Confident zeros**: Curated evidence of absence (retain as TN)
  - **Uncertain zeros**: Absence of evidence (mask out)
- Evaluate only on confident subset

Finding: All zeros in the clinical tensor have low confidence scores, meaning there is no curated evidence of absence. This strongly supports the PU framing interpretation.

D.5.4 Cohen's weighted kappa

For ordinal agreement between binned predictions and observations:

$$\kappa_w = \frac{p_o - p_e}{1 - p_e}$$

where p_o and p_e are weighted observed and expected agreement using linear weights:

$$w_{ij} = 1 - \frac{|i-j|}{n_{\text{bins}} - 1}$$

Interpretation scale: < 0 (less than chance), 0.0–0.2 (slight), 0.2–0.4 (fair), 0.4–0.6 (moderate), 0.6–0.8 (substantial), 0.8–1.0 (almost perfect).

D.6 Results summary

D.6.1 Dataset statistics

Metric	Value
Full tensor cells	17,329
Cells with mechanistic predictions	1,522 (8.8%)
Cells without predictions	15,807 (91.2%)
Known positives ($T_{\text{obs}} > 0$)	3,425 (19.8%)

bioRxiv preprint doi: https://doi.org/10.1101/2025.08.19.671142; this version posted December 30, 2025. The copyright holder for this preprint (which was not certified by peer review) is the author/funder, who has granted bioRxiv a license to display the preprint in perpetuity. It is made available under aCC-BY 4.0 International license.

D.6.2 Pathway dominance

Dominant Pathway	Count	Percentage
Barrier	890	58.5%
Latent	283	18.6%
Equal	349	22.9%

D.6.3 Overall performance (STRICT evaluation)

Metric	Value
Pearson r	0.349
Spearman ρ	0.299
Weighted κ	0.236 (Fair)
Recall	0.197
Specificity	0.974
Precision	0.421
F1	0.259
Balanced Accuracy	0.586

D.6.4 Per-archetype performance

Archetype	n	Pearson r	Spearman ρ	F1
Keystone	2,821	0.388	0.357	0.301
Opportunistic	6,045	0.355	0.316	0.248
Multi-host	4,030	0.333	0.305	0.219
Specialist	4,433	0.325	0.232	0.279

D.6.5 Enrichment analysis (PU framing)

Top K%	K	Positives Found	Precision@K	Enrichment
1%	173	51	0.29	2.9×
5%	866	256	0.30	2.9×
10%	1,733	508	0.29	2.9×
20%	3,466	870	0.25	2.5×

D.6.6 2D projection performance

Projection	Pearson r	Spearman ρ
PI (Pathogen × Immune)	0.359	0.374
PN (Pathogen × Niche)	0.583	0.555

D.7 S3.7 Interpretation of "predicted high, observed zero"

Cases where $\hat{T} > 0$ but $T_{\text{obs}} = 0$ are not necessarily model errors. They may indicate:

1. **Not studied**: The pathogen–niche–perturbation combination was never investigated
2. **Not detected**: Low statistical power or inappropriate study cohort
3. **No opportunity**: Pathogen not present in studied population

bioRxiv preprint doi: https://doi.org/10.1101/2025.08.19.671142; this version posted December 30, 2025. The copyright holder for this preprint (which was not certified by peer review) is the author/funder, who has granted bioRxiv a license to display the preprint in perpetuity. It is made available under aCC-BY 4.0 International license.

4. **True model miss**: Missing ecological constraints (temperature, microbiome, co-infections)

The finding that all clinical zeros have low confidence strongly supports interpretations 1–3 over interpretation 4.
